# Pathophysiology of Maternal Obesity and Hypertension in Pregnancy

**DOI:** 10.3390/jcdd12030091

**Published:** 2025-03-03

**Authors:** Joana Lourenço, Luís Guedes-Martins

**Affiliations:** 1 Instituto de Ciências Biomédicas Abel Salazar, University of Porto, 4050-313 Porto, Portugal; luis.guedes.martins@gmail.com; 2 Centro de Medicina Fetal, Medicina Fetal Porto—Centro Materno Infantil do Norte, 4099-001 Porto, Portugal; 3 Departamento da Mulher e da Medicina Reprodutiva, Serviço de Obstetrícia, Unidade Local de Saúde de Santo António EPE, Centro Materno Infantil do Norte, Largo Prof. Abel Salazar, 4099-001 Porto, Portugal; 4 Unidade de Investigação e Formação, Centro Materno Infantil do Norte, 4099-001 Porto, Portugal; 5 Instituto de Investigação e Inovação em Saúde, Universidade do Porto, 4200-319 Porto, Portugal

**Keywords:** maternal obesity, hypertensive disorders of pregnancy, pregnancy, hypertension, gestational hypertension, pre-eclampsia, eclampsia

## Abstract

Obesity is one of the biggest health problems in the 21st century and the leading health disorder amongst women of fertile age. Maternal obesity is associated with several adverse maternal and fetal outcomes. In this group of women, the risk for the development of hypertensive disorders of pregnancy (HDPs), such as gestational hypertension (GH) and pre-eclampsia (PE), is increased. In fact, there is a linear association between an increase in pre-pregnancy body mass index (BMI) and PE. Excessive weight gain during pregnancy is also related to the development of PE and GH. The role of obesity in the pathophysiology of HDP is complex and is most likely due to an interaction between several factors that cause a state of poor maternal cardiometabolic health. Adipokines seem to have a central role in HDP development, especially for PE. Hypoadiponectinemia, hyperleptinemia, insulin resistance (IR), and a proinflammatory state are metabolic disturbances related to PE pathogenesis, contributing to its development by inducing a state of maternal endothelial dysfunction. Hypertriglyceridemia is suggested to also be a part of the disease mechanisms of HDP. Therefore, this review seeks to explore the scientific literature to assess the complications of maternal obesity and its association with the development of HDP.

## 1. Introduction

Obesity is one of the biggest health problems in the 21st century and is the leading health disorder amongst women of fertile age, with a prevalence in this group that has been increasing during recent decades [1,2]. The statistics point to a global percentage of overweight or obese individuals of 39% [3]. Notwithstanding, the global prevalence of obesity is heterogeneous given the geographical, ethnical, and socioeconomical differences observed worldwide [4]. The global prevalence of obese and overweight pregnant women is inconsistent, but across the world, 39 million pregnancies are assumed to be associated with maternal obesity each year [5]. In fact, amongst all the women that become pregnant presently, it is estimated that half are obese or overweight [3]. Thus, obesity constitutes a serious risk to the health of the population, and it is the cause of great expenses for the healthcare system [3,6].

Obesity is usually diagnosed using body mass index (BMI), which is calculated by dividing the weight in kilograms by the height in meters squared (kg/m^2^) [1]. According to the World Health Organization (WHO), BMI may be subdivided into six categories: underweight (<18.5 kg/m^2^), normal weight (18.5–24.9 kg/m^2^), overweight (25.0–29.9 kg/m^2^), and obese class I (30.0–34.9 kg/m^2^), II (35.0–39.9 kg/m^2^), and III (>40 kg/m^2^) [1,2]. It should be noted that BMI has some limitations and does not take into consideration the distinct individual characteristics in physique and lean body mass, and, consequently, it is not the most reliable tool to reflect the distribution of body fat of an individual [2]. This classification, additionally, does not take into consideration the expected and rapid increase in weight during pregnancy, related to the augmented blood and extravascular volume, and the weight itself of the fetus and placenta [7]. New diagnostic tools have been proposed in recent years to overcome the disadvantages related with the use of BMI, namely the use of body fat percentage, waist/height ratio, waist-to-hip ratio, bioimpedance, and 3D scanning [8,9]. Despite its limitations, pre-pregnancy BMI is still the preferred method to identify pregnant women who are obese or overweight [7].

Maternal obesity poses a serious threat to both fetal and maternal health [3]. In fact, obesity is related with a higher risk of maternal cardiometabolic disease in the pregnancy and postpartum periods [10]. This cardiometabolic disfunction can manifest as the development of insulin resistance and gestational diabetes, hypertensive disorders, dyslipidemia, and CVD [10]. Hence, the pregnancy risks for the mother related to this condition are, amongst others, the development of hypertensive disorders such as pre-eclampsia (PE) and gestational hypertension (GH), gestational diabetes, preterm birth, emergency caesarean section, and intrapartum and postpartum hemorrhage [3,5,11]. The consequences for the offspring include a higher risk of developing metabolic syndrome, diabetes mellitus type 2 (T2DM), obesity, and cardiovascular disease (CVD) [3]. This suggests that an intergenerational transmission of metabolic dysfunction might be present, causing a vicious cycle of obesity and insulin resistance (IR) [6,11,12]. Therefore, the identification of obese women that are trying to conceive allows for the implementation of adequate pre-pregnancy medical counselling that can prevent the development of such complications, optimizing pregnancy and pediatric outcomes [2,3].

Hypertensive disorders of pregnancy (HDPs) constitute a broad term that comprise different entities such as PE/eclampsia, GH, chronic hypertension, and chronic hypertension with superimposed PE [13,14,15]. According to The International Society for the Study of Hypertension in Pregnancy, hypertension in pregnancy can be defined as a systolic blood pressure (sBP) greater than or equal to 140 mm Hg and/or a diastolic blood pressure (dBP) greater than or equal to 90 mm Hg in two separate measurements [3,14,16]. HDPs impact 8–10% of all pregnancies, and the worldwide prevalence of hypertension in women of childbearing age is increasing and is about 116 per 100,000 [13]. This increase in prevalence can be related to a higher number of obese and overweight women conceiving [3,17]. These hypertensive disorders are responsible for a great number of maternal deaths and are amongst the most common pathologies diagnosed in pregnant women [18]. As stated, one of the established risk factors for the development of hypertension during pregnancy, specifically GH and PE, is pre-pregnancy obesity, suggesting that there is a close relationship between these two conditions [2,3,16,18,19]. Excessive gestational weight gain (GWG) is also a risk factor for the development of HDP [20]. Ideally, GWG is determined by subtracting the weight at the first prenatal visit from the weight recorded at the final visit before delivery [20]. The use of reported pre-pregnancy weight to determine both total GWG and target GWG may be easier and is an equally valid strategy [20]. The Institute of Medicine guidelines recommend the appropriate GWG according to pre-pregnancy BMI: 12.5 to 18 kg for underweight, 11.5 to 16 kg for normal weight, 7 to 11.5 kg for overweight, and 5 to 9 kg for obese women [20].

The pathophysiological mechanisms linking maternal obesity and hypertension in pregnancy are still unclear [7]. The consensus appears to be that a complex maternal “obesogenic” environment characterized by systemic inflammation, IR, perturbances in adipokines, and metabolic dysfunction is the trigger for a series of pathological alterations in the placenta, leading to, for instance, an abnormal trophoblast invasion culminating in the development of PE [2,7,11,16]. This indicates that an excessive weight gain during pregnancy, or a state of obesity that precedes pregnancy, can predispose the mother to complications, such as PE and eclampsia, due to a dysfunctional metabolic and hormonal environment [18].

Most studies conducted focus on PE and on its physiopathology [3,18]. Some studies carried out in the last few decades have, in fact, demonstrated a relationship between a higher risk of PE and every increase in pre-pregnancy BMI [3,7]. However, it is still unknown to what extent the metabolic dysfunction seen in women with maternal obesity and hypertension in pregnancy is related to these conditions and is not a result of the metabolic changes induced by pregnancy itself [21]. Given the scarcity of studies carried out in recent years, the relationship between maternal BMI, on the one hand, and, on the other hand, the development of other disorders in this group, the metabolic pathways that lead to hypertension in pregnancy, and the molecular link between these two conditions, remains a subject that still needs to be elucidated [18]. Hence, this review aims to examine the current scientific literature from multiple perspectives to analyze the complications of maternal obesity and its relationship with the development of HDP.

## 2. Materials and Methods

To compose the present review, a search was carried out using the PubMed database. Pertinent keywords were used, such as “Maternal obesity”, “Pregnancy-induced hypertension”, “Pre-eclampsia”, “Eclampsia”, “Gestational Hypertension”, and “Hypertensive disorders of pregnancy”. These terms were used individually and in conjunction with one another.

To select the greatest possible number of articles related to the main theme of the review, we also used the Medical Subject Headings (MeSH) database through the PubMed’s Advanced Search Builder with the following MeSH terms: “Obesity, Maternal” AND “Hypertension, Pregnancy Induced” OR “Pre-Eclampsia”. In addition, the reference list of other studies with a similar objective to ours was analyzed for further articles that may be relevant in the context of our review.

For preselection, only articles written in English with a publication year posterior to 1995 and ones that were in accordance with the topic, as suggested by the title and abstract, were included. The complete text of these articles was then carefully analyzed to identify the greater number of studies with scientific rigor and relevance to our topic. Our search was repeated during multiple occasions to ensure that recently published studies were also analyzed and, if relevant, included in our review. In addition, we have composed this review based on recent guidelines such as: Hypertension in Pregnancy: Diagnosis, Blood Pressure Goals, and Pharmacotherapy: A Scientific Statement From the American Heart Association; The 2021 International Society for the Study of Hypertension in Pregnancy classification, diagnosis and management recommendations for international practice; The International Federation of Gynecology and Obstetrics (FIGO) Initiative on Pre-Eclampsia (PE): A Pragmatic Guide for First Trimester Screening and Prevention; and The Seventh Report of the Joint National Committee on Prevention, Detection, Evaluation, and Treatment of High Blood Pressure.

**Figure 1:**
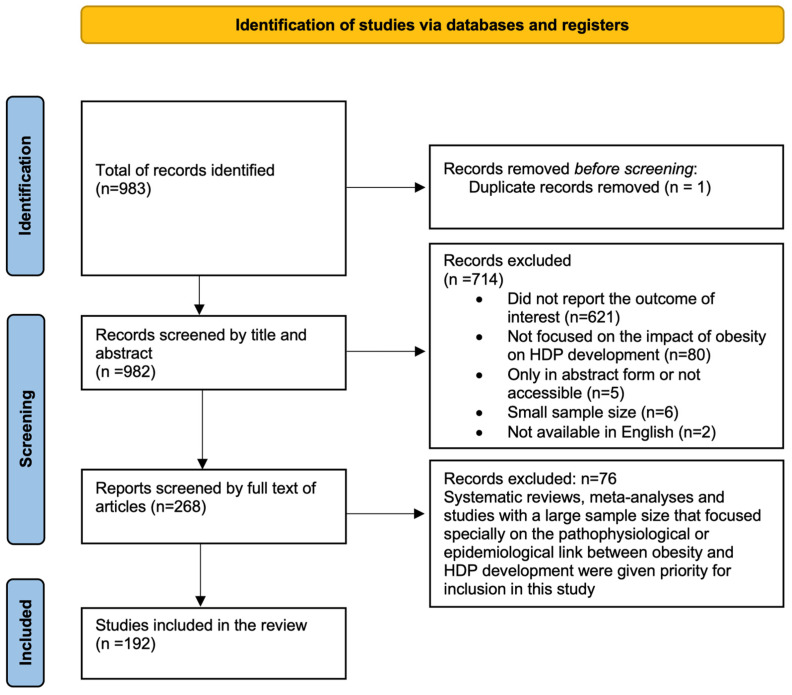
Flowchart of search results according to PRISMA 2020 guidelines.

The present review was conducted in compliance with the PRISMA 2020 guidelines. Thus, we obtained a total of 983 articles (after exclusion of one duplicated article) that contained the keywords stated previously or that were in the reference list of other studies that potentially would be of relevance for this review. After this selection, we advanced to the analysis of the full text of 268 articles; therefore, a total of 192 citations were finally included in our study (Figure 1).

## 3. Relation Between Obesity and Cardiovascular Risk Factors

Obesity is a complex disorder that is a result of diverse factors, including environmental, biological, behavioral, and socioeconomical factors [22]. These factors are interconnected with the economic conditions and gross domestic product of each country [23]. Globalization and the rising popularity of fast food have led to a uniform prevalence of obesity across Eastern and Western Europe and the United States, making it a significant global health issue [23]. Notwithstanding, obesity has slowly become a problem of not only the developed countries, specifically with a higher gross domestic product, but also of the developing countries, who have usually a lower gross domestic product [23]. This shift in obesity trends seems to reflect the fact that highly processed foods are usually cheaper and more easily available and, therefore, for countries with less economic prosperity the nutritional value of some foods can be deemed as secondary [23,24]. On the other hand, the fast-food availability and economical freedom associated with high income countries may help unravel the rising prevalence of obesity in these geographical areas [23]. Some populations may also face more cases of obesity due to genetic and metabolic predispositions, with this being an important biological factor to consider when addressing obesity numbers worldwide [25].

In recent years, many definitions of obesity have been proposed [23]. The WHO, for instance, states that obesity is characterized by an “abnormal or excessive fat accumulation that presents a risk to health” [23]. On the other hand, the World Obesity Federation proposes that obesity is a “chronic relapsing progressive disease” [23]. Regardless of its definition, obesity is usually diagnosed using BMI; however, this is a very narrow diagnostic standard, since it is not the best indicator of the patient’s cardiometabolic profile, and, therefore, its use as the primary diagnostic instrument for obesity is contested [26,27,28,29]. 

Recent evidence suggests that obesity is not a simple condition depending merely on BMI, but, in fact, encompasses a spectrum of different phenotypes [27,28]. These phenotypes—metabolically healthy overweight/obese, metabolically unhealthy overweight/obese, metabolic unhealthy normal weight, and sarcopenic obesity—differ in the location of adipose tissue (AT) accumulation, individual metabolic profile, and, ultimately, CVD risk [9,27,28]. New tools have been proposed to overcome these BMI limitations, such as the waist/height ratio, dual-energy X-ray absorptiometry, waist circumference (WC), waist-to-hip ratio, bioimpedance, and 3D scanning [8,9].

Obesity was once defined as a direct consequence of an excessive nutrient intake or positive caloric imbalance, low energy expenditure, and unhealthy dietary pattern [27]. However, currently, it is known that the former pathophysiological mechanism is too linear, and that obesity, being a heterogenous condition, emerges due to an interaction between a series of physiological factors and certain individual environments more prone to obesity [26]. These contributors act synergistically, creating a milieu that favors the accumulation of ectopic fat, the hypertrophy of adipocytes in the visceral AT, and the dysregulation of the AT itself [22,23,27]. This AT dysregulation, also referred to as adiposopathy, involves a series of anomalous endocrinological and immunological alterations that are responsible for the metabolic consequences of obesity [9,27]. These consequences are usually grouped in the term metabolic syndrome (MetS), representing the common association between obesity and a series of CVD risk factors such as hyperglycemia, arterial hypertension, hypertriglyceridemia, and decreased levels of HDL [9,30]. In fact, obesity is independently associated with the development of CVD, including heart failure, coronary artery disease, atrial fibrillation, and myocardial infarction [23,27,31]. This is a result of an excess in adiposity and subsequent adiposopathy, that can not only predispose the individual to a series of CVD risk factors but can also directly cause changes in the structure and function of the cardiovascular system [31].

The notion that AT exclusively constitutes a static storage for energy is long surpassed [23,32]. AT is, indeed, considered an active organ responsible for the secretion of cytokines, chemokines, and adipokines [23]. Adipokines constitute hormonal factors secreted by AT and are one of the primordial contributors to insulin sensitivity and to immunological, vascular, and angiogenesis homeostasis [23]. In addition, adipokines are responsible for controlling glucose and lipid metabolism [33]. Therefore, dysfunctional AT (adiposopathy) is the major key factor for the development of a dysmetabolic state of obesity and associated CVD risk factors [34].

In dysfunctional AT, there is an imbalance between proinflammatory and anti-inflammatory factors, specifically an increase in proinflammatory adipokines, such as IL-1 β, IL -6, IL-18, resistin, tumor necrosis factor α (TNF-α), and leptin, and a decrease in anti-inflammatory adipokines such as IL-10, secreted frizzled-related protein 5, nitric oxide (NO), and adiponectin, perpetuating an environment of low-grade chronic inflammation more prone to atherogenic repercussions [33,35,36]. Synergistically, in adiposopathy, the hypertrophic adipocytes are associated with a state of dysfunctional angiogenesis that altogether induce the necrosis and apoptosis of adipocytes [33,37]. This phenomenon stimulates the recruitment of activated macrophages, and a shift in normal local ones towards a proinflammatory phenotype (M1), creating, therefore, a vicious cycle of inflammation [33,37]. This chronic inflammation, combined with the hyperinsulinemia, hyperleptinemia and hypoadiponectinemia seen in obese individuals, is responsible for a state of impaired glucose signaling and IR and β-cell dysfunction and failure, culminating in the genesis of T2DM [23,33,36,38]. In fact, the relationship between T2DM and obesity is so close that the term diabesity has been proposed [35]. New studies have also been linking alterations in autophagy induced by obesity as one of the possible additional contributors to the development of T2DM [39].

Hyperleptinemia, leptin resistance, and hypoadiponectinemia are, in fact, amongst the crucial pathophysiological pillars for the development of not only IR but also of atherosclerosis and arterial hypertension [40]. Vasoconstriction, activation by leptin of the renin–angiotensin–aldosterone system (RAAS) and activation of the sympathetic nervous system (SNS), with subsequent renal sodium reabsorption, seem to account for the emergence of arterial hypertension [27]. Adiponectin seems to have a role in its physiopathology, since a case–control study conducted in 2004 demonstrated a link between hypertension and hypoadiponectinemia [41]. Within the scope of this study, a group of 446 male patients diagnosed with hypertension and 312 normotensive male patients were followed [41]. The results showed that a lower concentration of adiponectin was found in the group of hypertensive patients, and additionally, in the control group, adiponectin was found to be negatively correlated with blood pressure, which means that even in the control group, hypoadiponectinemia was associated with higher blood pressure levels [41]. Finally, a series of regression analyses confirmed that hypoadiponectinemia is, indeed, an independent risk factor for the development of hypertension [41].

**Figure 2:**
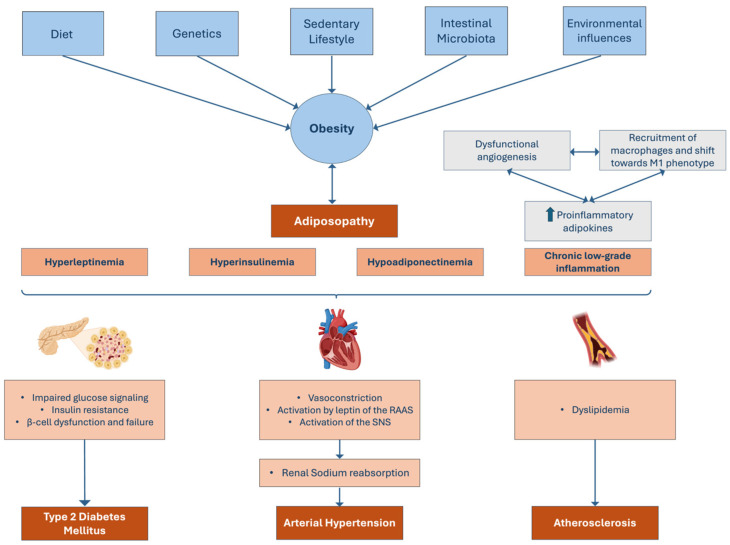
Obesity, as a multifactorial and heterogenous condition, is influenced by an individual’s diet, genetics, sedentary lifestyle, and intestinal microbiota, and by the environment itself. This “obesogenic” milieu favors the dysregulation of adipose tissue (AT) which is denominated as adiposopathy. Adiposopathy is characterized by hormonal imbalances, namely hyperleptinemia, hyperinsulinemia, and hypoadiponectinemia, and, additionally, by a state of chronic low-grade inflammation. The chronic low-grade inflammation seen in the AT of obese patients is a result of a dysfunctional angiogenesis, recruitment of macrophages and a shift in normal local ones towards a proinflammatory phenotype (M1), and, finally, of an imbalance between proinflammatory adipokines (IL1-β, IL-6, IL-18, leptin, TNF-α, and resistin) and anti-inflammatory adipokines (IL-10, secreted frizzled-related protein 5, NO, and adiponectin), favoring the proinflammatory ones. Finally, the chronic low-grade inflammation and the hormonal imbalances stated previously act synergistically, creating a dysmetabolic state which favors the development of Type 2 diabetes mellitus (T2DM), arterial hypertension and dyslipidemia, hence the metabolic syndrome (MetS) documented in most obese patients. Abbreviations: SNS—sympathetic nervous system, RAAS—renin–angiotensin–aldosterone system, TNF-α—tumor necrosis factor α, NO—Nitric oxide. Created with BioRender.com (version 04): https://BioRender.com/a81a039 (accessed on 22 November 2024).

Dyslipidemia is found in approximately 70% of obese patients [27]. This seems to be a result of an altered hepatic fat metabolism in which the rates of lipogenesis are augmented, contributing to an increase in the triglyceride pool, increased levels of low-density lipoproteins (LDLs) and decreased levels of high-density lipoproteins (HDLs) [42]. Finally, vascular dysfunction induced by the adiposopathy allied with the previously mentioned hormonal imbalances and dyslipidemia may also contribute to the coronary artery plaque buildup and development of coronary artery disease [27]. A study conducted in 2006 revealed, in fact, that low levels of adiponectin are associated with hypertriglyceridemia and, inversely, with low levels of HDL, additionally contributing to the overall dysmetabolic setting of obesity (Figure 2) [43].

This obesity-related adipose tissue disfunction, when present during pregnancy, can be the cause of programming of metabolic disease and obesity in the offspring, creating a vicious cycle characterized by an intergenerational transmission of these conditions [12]. Therefore, this state of adiposopathy seems to be the cause of an abnormal metabolic environment in utero that can predispose the fetus to metabolic dysfunction and CVD in later life [12,44]. One hypothesis is that a milieu of hyperglycemia and insulin resistance in utero, for instance, may predispose the fetus to the development of excess adiposity and increase the risk for the development of T2DM and prediabetes in the extrauterine life [44]. In fact, some studies have even suggested the role of maternal adiposopathy in the development of neurodevelopmental and cognitive disorders for the fetus in later life, enhancing the heterogeneous role of obesity as an important contributor to adverse pediatric and fetal outcomes [45]. Hence, obesity management and optimization of conditions such as T2DM in the pre-pregnancy period are fundamental strategies to improve not only maternal but also pediatric outcomes [45].

## 4. Obesity Management in Women of Reproductive Age

Obesity in women is related to a higher risk of adverse pregnancy outcomes such as hypertensive disorders of pregnancy, gestational diabetes, and stillbirth, amongst others [3,5]. Therefore, the management and counselling of obese women of reproductive age is crucial to prevent these complications and to improve pregnancy, maternal, and fetal outcomes [2,3]. The ideal time for the implementation of weight loss strategies is precisely in women of reproductive age, either as pre-pregnancy counselling or as an improvement in overall health [46]. In fact, prepartum and postpartum weight loss are regarded as safer approaches for minimizing the risk of adverse pregnancy outcomes [46,47]. Obesity is more likely to be diagnosed in female patients and the distribution of fat is different between female and male individuals; however, the role of gender is still often overlooked in the diagnosis and treatment of this disease [4].

The cornerstone of obesity management in female and male patients should consist, ideally, of lifestyle interventions since these are cheaper, safer, and less invasive methods contributing to a more organic weight loss process [48]. Nevertheless, according to the American Heart Association, the American College of Cardiology, and the Obesity Society, escalation of therapy, such as pharmacotherapy and bariatric surgery, is needed when modifications of lifestyle are not successful in producing the appropriate weight loss in obese patients [49]. Obesity management in adults, especially in women of reproductive age, is often an individualized and multidisciplinary approach which may include not only obstetricians and gynecologists but also general practitioners, dietitians, and psychologists [50].

Lifestyle interventions consist mainly of the modification of eating patterns or behaviors, as well as a reinforcement of physical activity aiming for a stable and moderate weight loss [48]. The two major guidelines of obesity management include one by the American College of Cardiology/American Heart Association/Obesity Society and a second by the American Association of Clinical Endocrinologists/American College of Endocrinology, and they remain the primary standard for addressing adult overweightness and obesity [50]. According to both guidelines, obese or overweight patients seeking weight loss should engage in aerobic exercise and resistance training allied with a calorie-restricted diet maintaining, therefore, an energy deficit to achieve the recommended weight loss [50]. The magnitude of weight loss should be individualized to account for the patient’s risk factors; however, in both protocols, it is stated that patients with obesity classes I and II should achieve a minimum weight reduction of 5% and, according to the latter guideline, that patients with significant comorbidities or complications should lose at least 10% of their body weight [51,52]. The recommended physical activity for most obese adults is a minimum of 150 min per week of moderate-intensity physical activity or between 75 and 150 min per week of high-intensity activity [53]. Resistance training two or three times a week has the additional benefit of preserving lean mass, such as muscle, during the weight loss process [54]. Furthermore, a set of behavioral interventions, such as target setting, dietary education, and sessions with clinicians, are recommended to promote adherence to the weight loss plan [50]. Therefore, ideally, obese women or overweight women with cardiometabolic disease should be encouraged to join intensive and holistic behavioral programs in the primary care setting [54].

Regarding the nutritional approach for obesity management, a reduced calorie intake (creating a deficit of 500 to 750 kcal per day, adjusted for personal body weight and activity level) is recommended to promote weight loss [54]. A vast group of diets have shown their benefits in the weight loss process, such as a Mediterranean-style diet, the Dietary Approaches to Stop Hypertension (DASH) diet, and a low-carbohydrate diet, and can, hence, be considered [54,55]. Additionally, control of portion size, minimizing ultra-processed foods (such as sugary beverages), and incorporating more fruits and vegetables into the diet constitute strategies that can minimize energy intake and support a sustained weight loss [54].

When lifestyle modifications fail to produce the desired weight loss, pharmacotherapy can be considered in non-pregnant female patients who follow adequate contraceptive use [49,54]. Anti-obesity medications are advised in adults with a BMI greater than or equal to 30 kg/m^2^ or adults with a BMI greater than or equal to 27 kg/m^2^ who present at least one obesity-related complication [56]. The medications for obesity management that are approved for long-term use in adults by the US Food and Drug Administration (FDA) and European Medicines Agency (EMA) include semaglutide and liraglutide administered subcutaneously, orlistat, and naltrexone/bupropion [54,56]. Moreover, phentermine, phentermine-topiramate ER, and tirzepatide have been approved for obesity management by the FDA [56]. There are innumerous additional medications which have been used off label, for instance, metformin, which can be beneficial for obese women with polycystic ovary syndrome [54]. The choice of pharmacotherapy should take into consideration the individual characteristics of each patient and the advantages of each anti-obesity medication, since some may be more beneficial than others; for example, GLP-1 receptor agonists such as liraglutide and semaglutide confer a greater improvement in glycemia, and thus they are expected to be the primary medication class for obesity management in patients with pre-diabetes [4,56].

Bariatric surgery can be considered for patients who present with a BMI equal to or greater than 40 kg/m^2^ and those with a BMI between 35 and 40 kg/m^2^ who present with other comorbidities [54,57]. Patient selection criteria encompass the BMI standards outlined above, as well as an unsuccessful medical therapy [57]. The most common surgical procedures include the Roux-en-Y gastric bypass, sleeve gastrectomy, biliopancreatic diversion with duodenal switch, and adjustable gastric banding, with the first two being the preferred methods [54,57]. A study published in 2020 suggested that the benefits of bariatric surgery in obese women go beyond the cardiometabolic benefits and hold potential for managing multiple disorders, including hormone-related disorders, in this group of patients [58]. In fact, bariatric surgery seems to have the ability to improve the cardiometabolic dysfunction associated with obesity, with evidence suggesting an improvement in glycemic, lipidic, and tensional levels [59]. The overall obesity-related risk of cardiovascular and cerebrovascular disease seems to also be reduced after bariatric surgery [59]. Nonetheless, bariatric surgery poses some risks that should not be overlooked [60]. Micronutritional deficiencies such as iron, folic acid, vitamin B12, vitamin A, and vitamin C are possible and common complications that should motivate a closer monitoring [60]. If present, adequate supplementation should be instituted [59]. These surgery procedures may also cause profound endocrine changes and affect the somatotropic, gonadal, thyroid, pancreatic, and adrenal systems that may be of clinical relevance [61]. Therefore, although bariatric surgery seems to be an efficient option for the management of obesity, its risks and limitations should be taken into consideration when deciding the best individualized approach to obesity [61].

It should be noted that in women of reproductive age, the use of combined hormonal contraceptives may be associated with weight gain and thromboembolic events [62]. Therefore, their use in obese women ought to be carried out with caution and, consequently, long-acting and reversible contraception such as progestin-only implants, copper IUDs, IUDs with release of levonorgestrel, and progestin-only methods are considered safer contraceptives for obese women within the reproductive age range [62].

## 5. Management of Pre-Pregnancy, Pregnancy, and Postpartum Obesity

Gynecologists and obstetricians assume a unique role by having contact with women in several life phases and can, therefore, impact the obesity risk and its prevalence through the promotion of lifestyle modifications and by instituting the appropriate therapy in obese women before pregnancy, after pregnancy, and during the pregnancy period [63]. The perfect time for the implementation of lifestyle modifications and obesity management for women is, in fact, during the pre-pregnancy phase, including the preconception period, or during the postpartum phase, including the interpregnancy period, and this implementation must be reinforced by clinicians [46,47,63]. Weight loss may not be recommended during the pregnancy period given the fact that a possible consequence of weight loss may be a higher risk of small-for-gestational-age babies; this further highlights the great importance of pre-pregnancy and postpartum weight loss [1,47]. However, several studies conducted since 2009 have documented that in women who lost weight during pregnancy or who did not meet the expected GWG, the maternal outcomes were indeed optimized [20]. Practices for managing maternal obesity may differ worldwide because innumerous international guidelines have been proposed in recent years [64]. However, the International Federation of Gynecology and Obstetrics (FIGO) created a guideline in 2020 to aid clinicians in the management of obesity for women during their different life phases [63].

The preconception period is particularly important for weight management, since during this period, it is possible to minimize not only the risks of noncommunicable diseases (NCDs) for the mother but also the intergenerational transmission of these NCDs to the offspring [65]. It should be noted that even small reductions in pre-pregnancy weight are associated with better fetal and maternal outcomes [1,63]. A lower risk of PE, restoration of the menstrual cycle, and improvement in fertility have been associated with a loss of 5 to 10% of the patient’s body weight [2]. According to the FIGO guideline, during contact with primary care services, all women of reproductive age should have a BMI assessment and, in the case of obese women, should receive counselling on their weight and on possible advantageous lifestyle modifications [63,66]. Obese women may not acknowledge their weight or unhealthy habits, and their personal knowledge of the risks that obesity carries can be limited [67,68,69]. Consequently, women of reproductive age should receive advice on the risks associated with obesity, the impact of obesity on the patient’s fertility, and adverse effects on pregnancy and childbirth that are related to obesity [63,70]. Lifestyle modifications consisting of a healthy diet and physical exercise should be the first-line option for women in the pre-pregnancy period, and a realistic weight loss target should be set, which generally is the loss of 5 to 10% of the patient’s body weight during a semester [1,63]. Pharmacotherapy is not recommended during pregnancy or during the preconception period for women who desire a pregnancy in the short term, given its possible side effects and safety concerns, but it should be recommended as part of the management program for women with more severe obesity or with comorbidities who desire to become pregnant in the long term [1,63]. Medications such as GLP-1 agonists, metformin, orlistat, and naltrexone/bupropion are possible therapies for the management of obesity approved by the EMA and FDA [52]. Bariatric surgery can be considered in women with obesity class III and class II who present with multimorbidity; however, given the risk of the surgery-related nutritional deficiencies and possible related adverse perinatal outcomes, pregnancy should be postponed for at least 12 to 18 months post-surgery [2,63,71]. To prevent pregnancy during this period, effective contraception should be implemented [72]. Bariatric surgery, specifically Roux-en-Y gastric bypass, is associated with a lower risk of HDP [73]. Additionally, obese women with a BMI greater than or equal to 30 kg/m^2^ who are planning to conceive should be counseled to escalate the dose of daily folic acid supplementation to 4–5 mg, starting at least 1–3 months prior to conception and continuing into the first trimester [63,65,72,74]. This a consequence of the fact that obese women have a higher risk of neural tube defects [63,65].

During their first antenatal visit, all pregnant women should have their BMI measured to identify obesity or overweightness and to further classify associated pregnancy risks [63]. During antenatal care visits, monitoring and management of GWG is recommended given that obese pregnant women who have a BMI equal to or greater than 30 kg/m^2^ should be advised to limit their GWG to between 5 and 9 kg, according to the Institute of Medicine guidelines [1,63]. Similarly to the pre-pregnancy management of obesity, pregnant women should be informed about the labor-related risks and the impact of obesity on maternal and fetal health [63]. Obesity management during pregnancy is based almost solely on dietary control and on exercise prescription, and during this phase, women may be more receptive to said interventions [63,70,75]. There is no need for alteration of a healthy pre-pregnancy diet, but patients should be encouraged to consume nutrient-dense foods [76]. According to the FIGO’s recommendations on maternal nutrition, obese women should only slightly increase their energy intake during the last 3 months of their gestation [77]. Physical activity during pregnancy has been linked to a lower risk of pregnancy-related complications, such as HDP, and should be recommended for a minimum of 150 min per week, or 30 to 60 min daily for most of the days of the week, and should consist of moderate-intensity exercise [10,63,78,79,80]. The type of activity should be individualized with each patient, considering their previous physical activity, and it may be a combination of aerobic and anaerobic exercise [63,75,77,81]. However, high-intensity physical activity should be avoided during the third trimester [63,75,77,81]. Vitamin D supplementation may also be considered, and for women who have undergone bariatric surgery, close nutritional follow-up must be instituted [63,70]. Some obese women may also benefit from the PE prophylaxis with aspirin in the 150 mg dose daily when other risk factors for this condition are present, e.g., for women who are more than 40 years of age, for primipara mothers, for mothers with multiple gestations, and for women with a history of PE in the family [1,63,70,82,83]. Some studies also suggest the use of an incremental dose of aspirin, such as 162 mg, in obese pregnant women due to altered pharmacokinetics in these patients [84,85]. Additionally, all obese pregnant women with a BMI equal to or greater than 30 kg/m^2^ should have a gestational diabetes screening in the initial stages of pregnancy [63]. In the first trimester, screening for PE using mean arterial pressure may be advantageous, and the FIGO recommends the additional use of placental growth factor (PIGF) or plasma protein A in tandem with monitoring of the uterine artery doppler index, although further evidence is needed to confirm the applicability of these biomarkers and to clarify their role in enhancing the early screening of preterm PE at this stage [63,83,86,87,88,89,90].

Labor for obese women may have some particularities, and thus, all pregnant women should have an individual assessment of their mode of delivery [63]. For women with class III obesity, an anesthesiologist consultation may also be prudent given the higher risk of anesthesia-related complications [63]. Maternal obesity is associated with a higher risk of emergency caesarean section [63,91,92]. Nonetheless, maternal obesity is not a strict indication for its use, and hence, cesarean delivery is reserved for the standard obstetric criteria [91]. In some cases, induction of labor at 41 weeks of gestation may be an option for women who present a BMI equal to or greater than 35 kg/m^2^, to reduce the risk of adverse perinatal outcomes, such as fetal death, and to reduce the likelihood that a caesarean section will be required [63]. For class III obese women who undergo vaginal delivery, prompt insertion of an epidural catheter and venous access may be adequate in the eventuality of an emergent caesarean section or other interventions [63].

Regarding postpartum obesity, the reinforcement of the positive role of breastfeeding and support for its initiation should be present during the antenatal and postnatal visits given its benefits not only for the child but also for the mother, including lower postpartum weight retention [63]. Obese women who have been diagnosed with pregnancy complications such as PE or gestational diabetes should receive adequate postnatal follow-up given the close relationship between these complications and CVD in later life [63]. Weight loss in the interpregnancy period has been associated with a lower risk of HDP, gestational diabetes, and stillbirth in subsequent pregnancies [2,63]. Weight loss or, if not possible, weight maintenance, with adequate interventions, should be encouraged in postpartum obese women, since postpartum weight, especially during the first year after pregnancy, may influence women’s long-term BMI [63]. During the postpartum period, inadequate nutritional consumption is the mainstay of weight retention, and therefore nutritional counseling allied with exercise are the pillars of the obesity management approach [2,63]. Finally, contraception, if in line with the patient’s will, should be initiated to prevent unwanted pregnancies given the several risks associated with maternal obesity [63].

## 6. Impact of Obesity on Hypertensive Disorders of Pregnancy

HDPs are relatively frequent amongst pregnant women, and their role as an important determinant of adverse fetal and maternal outcomes is clear [2,7,93,94,95,96,97]. The definition of these disorders is nonconsensual and varies between countries and guidelines; however, generically, it includes a group of four conditions: PE/eclampsia, GH, chronic hypertension, and chronic hypertension with superimposed PE [15]. Chronic hypertension is defined as high blood pressure (sBP greater than or equal to 140 mm Hg and/or a dBP greater than or equal to 90 mm Hg) present in women before pregnancy or diagnosed until the 20th gestational week, and GH is a condition characterized by isolated high blood pressure diagnosed after the 20th week of pregnancy [15,93]. On the other hand, PE is a state of high blood pressure present de novo, after the 20th week of gestation associated with proteinuria (>300 mg within 24 h), organ dysfunction, or placental insufficiency [15]. Eclampsia corresponds to the cerebral involvement in PE which clinically translates to seizure development [98]. Chronic hypertension with superimposed PE is characterized by the presence of proteinuria or organ damage in a patient previously diagnosed with chronic hypertension [15]. HELLP syndrome is an entity that remains widely regarded as a manifestation of severe PE and it is defined by the phenomenon of hemolysis, alongside an increase in hepatic enzyme levels and a reduction in the platelet count [99].

The majority of studies conducted during the last few years focus particularly on the relationship between pre-pregnancy and pregnancy BMI and the emergence of hypertensive disorders, especially PE, during gestation [100]. Other indicators of a maternal dysmetabolic or obese state, such as WC, percentage of body fat, and waist-to-hip ratio, amongst others, have been proposed as substitutes for BMI as an absolute risk indicator for HDP, and, in fact, a recent study published in 2024 explored the relationship between these factors and the development of pregnancy-induced hypertension (PIH) through an extensive meta-analysis and Mendelian randomization [100].

Obesity is a well-known risk factor for the development of HDP, and this knowledge is due to the numerous studies and research endeavors undertaken [95,100,101,102,103,104,105,106,107,108,109,110,111,112]. In fact, a study conducted in 2015 established that an increase in BMI is associated with a rise in mean arterial pressure in normotensive women and in women with chronic hypertension [113]. Pre-pregnancy BMI was found to be independently associated with increased levels of blood pressure and with the development of HDP in studies conducted in 2016 and 2012, respectively [114,115]. Since then, several studies worldwide have shown the close relationship between obesity and HDP in specific populations, with some stating that the risk of developing PIH is three times higher amongst obese women [97,101,116,117,118,119,120]. Moreover, a study published in 2003 determined that the risk of developing PE in women doubled with every five-to-seven unit increase in BMI, demonstrating an exponential relationship between these two conditions [121]. In fact, meta-analyses have elucidated a dose-response correlation between the classifications of obesity and the incidence of PE, indicating that the risk is elevated by a factor of three to four for individuals categorized with obesity class II or III in comparison to those with class I [2]. This exponential relationship between each obesity class and HDP risk was also demonstrated in a retrospective cohort study conducted in 2024 [109]. A study published in 2019 regarding Portuguese pregnant women confirmed that obesity is related to a higher risk of developing PIH in this population; this finding is accordance with observations in other countries [122]. Obese mothers have an increased risk of developing essential hypertension before pregnancy and, therefore, of carrying this condition into pregnancy [123,124,125]. Obesity has also been associated with the progression of hypertension during gestation and, consequently, with more severe states of hypertension, such as PE [126]. An elevated pre-conception BMI was similarly associated with an increased likelihood of developing HELLP syndrome [99].

Two studies published during 2024 concluded that pre-pregnancy obesity or overweightness is not only related to a higher risk of PE but also with an increased risk of developing GH [100,127]. This was an association already shown in other studies conducted in previous years which demonstrated that BMI is an independent risk factor for GH and PE development [114,128]. In addition, WC was also shown to have a relationship with HDP, with higher levels in WC being related to an increased overall risk of PE, as shown by a meta-analysis and Mendelian randomization conducted by the first author mentioned [100]. A study conducted in 2022 and one conducted in 2013 seem to support this finding by having shown that higher levels of WC and BMI were related to a higher risk of GH and PE [129,130]. The Mendelian randomization study previously mentioned also revealed that BMI is not the only parameter related to PIH, and in fact, in this study, for every standard deviation increase in BMI, WC, body fat percentage, hip circumference, and other obesity parameters, an increase in GH and PE risk was observed [100]. Furthermore, WC was found to be a superior parameter regarding the estimation of the risk for GH and PE, and, as it is a simple and easy measurement capable of inferring visceral adiposity and HDP risk, it should be considered as a valuable clinical resource when evaluating obese women [100]. In a study published in 2008, associations between neck circumference and risk of developing PE and PIH, and between self-reported snoring and risk of developing PE were found [131]. These markers may also prove to be of clinical significance when approaching obese patients [131].

A high GWG, especially during the first trimester, seems to be, additionally, an independent risk factor for HDP, specifically for GH and PE [128,132,133,134]. Furthermore, women with a high pre-pregnancy BMI seem to have a higher likelihood of experiencing a GWG above that recommended by the guidelines, although some studies have found a reverse association [128,135,136,137]. The combined risk for PIH that accompanies obesity and high GWG makes obese women with an excessive GWG the ones at highest risk for adverse pregnancy outcomes, including PIH and, specifically, PE [138,139,140,141]. This should alert the clinician to the potential risks of not only obesity itself but also of the GWG in obese pregnant women, and, therefore, should prompt closer monitoring of the patient’s weight during gestation for women in this group [139,142].

The pathophysiological link between obesity and HDP remains to be clarified, but, nonetheless, several theories have arisen in recent years [100]. Obesity is, generically, a proinflammatory condition associated with the systemic release of proinflammatory factors and free fatty acids [23,40,100]. Therefore, obesity is frequently associated with hypertriglyceridemia, T2DM, and dyslipidemia [26,32]. The pathophysiology between obesity and HDP is complex; however, a plausible mechanism of disease is based on the fact that this obese dysmetabolic state may alter the comprehensive responsiveness of maternal endothelial cells to circulating mediators, such as those released by the placenta, making the mother more susceptible to the development of PIH [100]. A proinflammatory environment and IR associated with obesity are factors that have been linked to the development of HDP and, especially, to the development of PE [3]. Decreased levels of atrial natriuretic peptide, hyperinsulinemia, and increased sodium reabsorption act synergistically to increase water retention and promote hypertension in obese women [143]. High estrogen levels, mediated by the accumulation of AT in obese women, have also been linked with sodium retention trough an aldosterone-dependent pathway [144]. On the other hand, the hyperleptinemia, hypoadiponectinemia, and overall state of oxidative stress that are seen in obesity are responsible for inducing endothelial dysfunction, contributing to the systemic organ lesions observed, for instance, in PE [3,143]. Hyperleptinemia has been, in fact, linked with the development of GH and PE due to its proinflammatory effects and to the endothelial dysfunction that it can stimulate [3,145].

**Figure 3:**
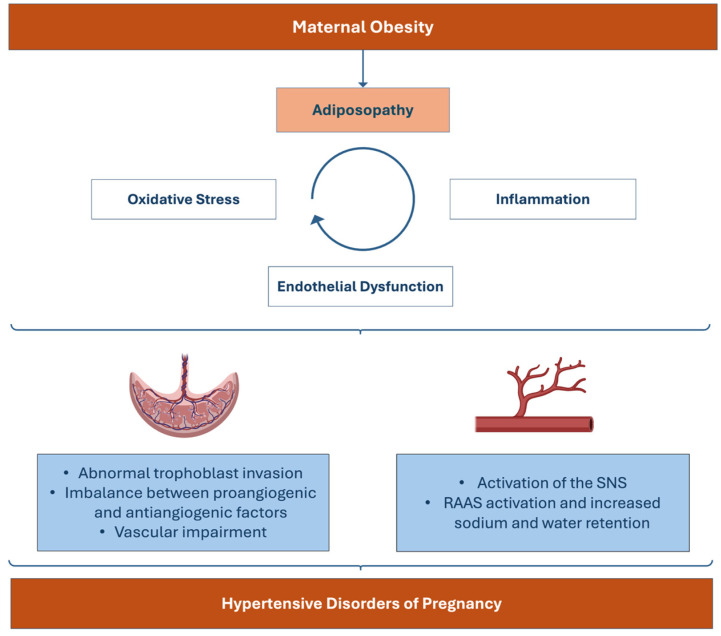
Maternal obesity is an emerging condition characterized by a state of profound dysfunction of the AT allied with the consequential dysregulation of the immunological and hormonal systems. This state is frequently referred to as adiposopathy. Adiposopathy includes various metabolic alterations, such as hyperleptinemia, hypoadiponectinemia, dyslipidemia, hyperinsulinemia, and IR, that altogether induce an environment of endothelial dysfunction, oxidative stress, and systemic low-grade inflammation. These alterations seem to act synergistically, affecting the normal trophoblast invasion process and perpetuating a state of imbalance between proangiogenic and antiangiogenic factors, with a consequential state of vascular impairment. This may partially explain the pathophysiological relationship between PE and obesity. Moreover, this obesogenic environment seems to be responsible for the activation of the SNS and of the RAAS, with consequential sodium and water reabsorption and development of hypertension. This mechanism may be behind the development of other hypertensive disorders of pregnancy, such as gestational hypertension (GH) and chronic hypertension. Created with BioRender.com: https://BioRender.com/e21l96 (accessed on 15 January 2025).

Hypertriglyceridemia may develop during pregnancy as a consequence of an elevated IR and the presence of estrogen and may not be a direct consequence of obesity itself, but irrespective of the cause, increased levels of triglycerides have been associated with an increased risk of HDP [100]. The first study mentioned also confirmed the mediating role of triglycerides on HDP development, emphasizing its clinical significance [100]. This is particularly noteworthy, as hypertriglyceridemia can sometimes precede the onset of these conditions, offering potential predictive value [100]. These disorders have a complex pathophysiology, and several maternal factors act synergistically and contribute to its development [15,146]. Hence, the notion that several maternal pathologies coexist and act together to contribute to an increased risk for the development of HDP is essential [146]. For instance, obesity is often associated with a state of IR and TDM2, and the risk for the development of PE is higher when these two conditions are present compared with the risk that either one poses individually (Figure 3) [146].

Hence, obesity management is of maximum importance to prevent the aforementioned complications, with the benefits of diet and physical activity on GH and PE being documented by a series of systematic reviews and meta-analyses [85,147,148,149,150]. One of the systematic reviews and meta-analyses mentioned previously documented a reduced risk of GH, specifically, with the implementation of aerobic exercise during pregnancy [148]. Lifestyle modifications during pregnancy have the capacity for reducing the risk of excessive GWG and postpartum weight retention, which is important not only in reducing the risk of PIH in the present pregnancy but also for minimizing the said risk in future gestations [147,151].

The treatment of hypertension in the pregnancy period has been marked by several international guidelines that suggest not only different thresholds for the initiation of antihypertensive medications but also different blood pressure targets [10]. It should be noted that antihypertensive agents may be associated with small-for-gestational-age babies and, therefore, its use must be cautious and, when possible, avoided [152]. For instance, according to The Seventh Report of the Joint National Committee on Prevention, Detection, Evaluation, and Treatment of High Blood Pressure guidelines, for women with stage 1 chronic hypertension, medication may be avoided and lifestyle modifications should be instituted as first line therapy [152]. However, pregnant women with target organ damage or a history of requiring multiple antihypertensive agents for blood pressure control should continue antihypertensive medication as needed to maintain adequate blood pressure levels [152]. The American College of Obstetricians and Gynecologists recommends antihypertensive therapy for women with pre-eclampsia who have a sustained sBP of ≥160 mmHg or dBP of ≥110 mmHg, as well as for those with chronic hypertension at the same thresholds [85,153]. Severe hypertension (sBP of ≥160 mmHg or dBP of ≥110 mmHg) in pregnancy should motivate urgent antihypertensive treatment in a controlled setting [85]. The target treatment range recommended by this society is 120 to 160/80 to 110 mmHg [153]. Internationally, most hypertension societies advocate for a more aggressive approach, recommending antihypertensive treatment when blood pressure reaches ≥140/90 mmHg [153]. The first-line agents for the treatment of non-severe hypertension in pregnancy are oral drugs such as labetalol, methyldopa or nifedipine [85]. Severe hypertension should be treated with oral nifedipine or labetalol or with IV agents such as labetalol or hydralazine [85]. Women with eclampsia or with pre-eclampsia and neurological signs or symptoms or with pre-eclampsia and severe hypertension should receive magnesium sulphate [85]. Timing of delivery varies and should be individualized considering the subtype of HDP, gestational age, maternal signs and symptoms, and with the risk of maternal or perinatal death [85].

## 7. The Role of Obesity in Pre-Eclampsia Pathogenesis

PE is a condition that affects 3 to 8% of the total number of pregnancies worldwide [143]. PE is characterized by hypertension de novo diagnosed after the 20th gestational week combined with signs of end-organ failure or insufficiency, such as proteinuria (≥0.3 g in 24 h or ≥1+ proteinuria), thrombocytopenia (platelet count of <100,000/μL), renal insufficiency (a creatinine concentration of >1.1 mg/dL or double its serum concentration), altered liver function, pulmonary edema, or signs of altered neurological function (visual symptoms, for instance) [3,83]. Several risk factors for the development of this pathology have been determined and arbitrarily categorized as high or moderate risk factors [143]. Classically, obesity has been labeled as a moderate risk factor for PE development, but recent studies have determined the increasingly central role of obesity on the pathophysiology of this condition [143,154]. PE can be further subdivided into two categories according to gestational age, namely early-onset PE when the condition is present until the 34th gestational week and late-onset PE when this condition emerges after the 34th week of gestation [155]. Additionally, the existence of two different subtypes of PE, namely a hypovolemic, “classic”, or placental PE and a hypervolemic, maternal, or term PE, have been described [143,156]. Late-onset PE is characteristically associated with primiparity and obesity, and this seems to account for the higher prevalence of late-onset PE in developed countries given the parallel and increasingly higher prevalence of obesity [157]. In fact, both an excessive GWG and a BMI greater than 30 kg/m^2^ are conditions related to the emergence of late-onset PE [143].

PE is proposed to be the result of an abnormal trophoblast invasion and a sequential abnormal remodeling process of the spiral arteries which leads to a placental environment characterized by hypoxia and oxidative stress [3,18,98,158,159]. This hypoxic placental state seems to be responsible for the release into the maternal circulation of antiangiogenic factors, such as soluble fms-like tyrosine kinase-1 (sFlt-1), and proinflammatory factors, such as TNF-α, with the consequential development of signs and symptoms of end-organ damage [3,106,160]. This placental release of soluble factors seems to cause a generalized endothelial dysfunction and, consequently, an increase in systemic vascular resistance and maternal hypertension [98,153,159]. Decreased levels of NO and increased levels of endothelin seem to be responsible for this endothelial dysfunction [161]. Indeed, obesity and PE share many characteristics and have similar metabolic disturbances, suggesting an overlap between these two conditions [18,21]. Some studies based on animal models have reported PE-like symptoms in obese mice, with parallel evidence of alterations in the placental function [162,163]. It is posited that the association between maternal obesity and PE is due to the role of immune cells residing in the maternal AT, as well as in the placenta, and the adipokines released by the AT, which may lead to a compromised placentation process and the consequent disease manifestations [164,165].

An imbalance between proangiogenic factors, such as PIGF and vascular endothelial growth factor (VEGF), and antiangiogenic factors, such as sFlt-1 and soluble endoglin, has been proposed as one of the key pathological alterations in PE, since it may produce a state of placental ischemia and precede the development of symptoms in pre-eclamptic patients [98,153,166,167]. In fact, a study conducted in 2024 evaluated the presence of the said angiogenic abnormalities in different subgroups of patients, specifically patients with preterm and term PE [156]. The angiogenic profile of each woman was evaluated through a plasma ratio of PIGF and sFlt-1 [98,156]. The results demonstrated that in the late-onset PE group, only half of the women presented an alteration in angiogenic profile, contrasting with the fact that almost every woman in the early-onset PE group presented the aforementioned alterations [156]. These findings seem to suggest that additional disease mechanisms may be present in the first group of patients, corroborating the conclusion that the pathophysiology of the two PE subtypes is intrinsically different [156]. Indeed, it is believed that the development of the late-onset PE subtype is a result of complex interactions between innumerous pre-existent conditions that indicate a poor maternal cardiometabolic health, such as obesity, IR, and T2DM [166]. Notwithstanding, obesity seems to act by aggravating the angiogenic imbalance previously mentioned, since an exaggerated release of sFlt-1 from the AT, the placenta, and circulation has been shown in several animal and human studies [161,165,167,168]. PIGF levels are presumed to be decreased in obese individuals; this was confirmed in two animal model studies conducted in 2020 and 2010 which demonstrated that the recombinant administration of PIGF and VEGF, respectively, had the ability to reduce blood pressure in vivo in hypertensive pregnant obese rats [161,169,170]. These findings seem to strengthen the idea that decreased levels of proangiogenic factors assume a key role in PE development for both subtypes [161,169].

Obesity is indeed related to a dysmetabolic state of profound dysfunction of the endocrinological and immunological systems [3]. Adipokines are released by the obesogenic environment and assume a central role in determining the dysmetabolic setting of obesity, seeming to be catalysts for HDP development [3,145,167,171,172]. Altogether, hyperleptinemia, hyperinsulinemia, hypoadiponectinemia, and hyperlipidemia are suggested to have the capacity for contributing to the overall hypoxic and dysfunctional state of the placenta in obese pregnant women, leading to the emergence of PE [3,18,161,164]. Hypoadiponectinemia has been related to IR in obese individuals, and although there is lack of consensus in the literature regarding whether its levels are decreased in cases of PE, it seems to be an important contributor to PE development [3,18,145]. Hypoadiponectinemia and hyperleptinemia combined with the oxidative stress present in obese patients promote an inflammatory state and an environment prone to endothelial dysfunction [3,143,173,174]. This endothelial dysfunction seems to be related to a decreased bioavailability and synthesis of NO induced by the increased levels of leptin and decreased levels of adiponectin [18]. The decreased levels of NO may also help explain the development of PE observed in obese patients due to the increased vascular dependence of NO to achieve vasodilation in obesity, as suggested by a study conducted in 2013 on obese melanocortin-4 receptor- deficient rats [175]. Hyperleptinemia, moreover, has been related to the creation of a thrombosis prone setting through an enhancement of platelet aggregation [3]. Additionally, a study conducted in 2013 using animal models demonstrated a direct effect of hyperleptinemia on blood pressure through an aldosterone-related mechanism [3,174]. Elevated levels of leptin were also associated with an abnormal cytotrophoblast differentiation, thus contributing to the hypoxic placental setting associated with PE [18,161,168]. Interestingly, the effects of leptin on blood pressure have been proposed to be aggravated by the hyperestrogenemic milieu of pregnancy [161].

Hyperinsulinemia is associated with increased water and sodium retention and, consequently, the development of hypertension [143,165]. Moreover, insulin seems to have a role in disrupting the normal synthesis of NO, contributing to the generalized endothelial dysfunction previously stated and to a consequential state of impaired vasodilation [143,165]. A study conducted on obese mice in 2023 did, in fact, uncover a major role of insulin in the development of PE [176]. In this study, it was demonstrated that insulin can interfere as early as in the trophoblast differentiation process via a molecular dependent pathway, specifically by perturbing the degradation of the inhibitor of DNA binding protein 2, whose levels corelate with a normal placentation process [165,176]. Inositol phosphoglycans (IPGs) are second messengers of insulin [166]. IPGs of lipidic form released by the fetal or placental bed have the capacity of inducing a proinflammatory response in the maternal environment through leakage of these substances into the maternal circulation [166]. These molecules may, hence, aggravate and contribute to the state of endothelial dysfunction seen in most patients with PE and hyperinsulinemia or IR [143,166]. Moreover, elevated levels of molecules that increase blood viscosity, such as profibrinogen and plasminogen activator inhibitor 1, and molecules that enhance reabsorption of sodium in the kidneys, such as angiotensinogen, in obese patients may contribute to the overall hypertensive state during pregnancy and to the development of HDP such as late-onset PE [143,167]. In addition, agonistic autoantibodies to the angiotensin II type 1 receptor have been shown to be produced by the placenta of pre-eclamptic patients, and their action of elevating blood pressure may be aggravated in obese individuals [18,177].

The systemic low-grade inflammation state of obesity is also of great importance for PE pathogenesis [3,18,178,179]. This proinflammatory state is related to IR and with the induction of endothelial dysfunction, and it may act as the link between obesity, IR and T2DM, on the one hand, and the development of GH and PE, as suggested by some authors [3,18,180]. The proinflammatory cytokines released in obesity, that is, IL-6 and TNF-α, amongst other molecules such as C-reactive protein, may enhance the state of IR and endothelial dysfunction through the maximization of the cellular oxidative stress [3,181]. These inflammatory cytokines have also been reported to increase the levels of sFlt1 and may explain its increased levels in obese individuals [98]. It should be noted, however, that some studies have not reported higher levels of TNF-α in obese, pregnant individuals when compared with pregnant, non-obese individuals [181]. Moreover, in obesity, this proinflammatory environment is enhanced—via an increased activation of the alternative complement pathway, involving the family of toll-like receptors 4—by the increased levels of C3 seen in some obese individuals [3,164,182]. Increased levels of C3, released by the AT, can also be responsible for a shift in the balance between proangiogenic and antiangiogenic factors, favoring the latter and contributing to a hypoxic placental state [164].

**Figure 4:**
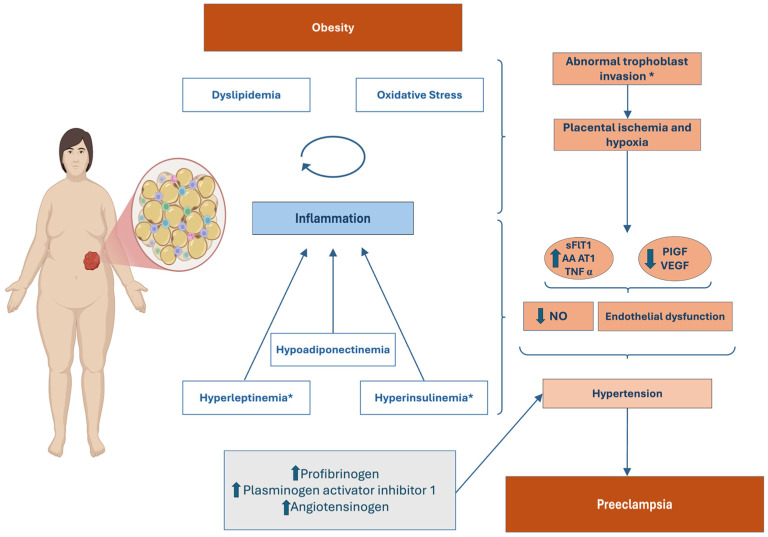
This figure aims to demonstrate the possible relationship between the metabolic disturbances associated with obesity and the development of pre-eclampsia (PE). PE seems to be the result of complex interactions between the fetal and maternal environments. Indeed, it is believed that this condition is caused by an abnormal trophoblast invasion and an inadequate remodeling process of the spiral arteries of the uterus, leading to a reduced uteroplacental perfusion and, consequently, hypoxia and ischemia of the placenta. The distressed and hypoxic placenta releases antiangiogenic factors, such as soluble fms-like tyrosine kinase-1 (sFlt-1), and proinflammatory factors, such as TNF-α, into the maternal circulation. Simultaneously, decreased levels of angiogenic factors, such as placental growth factor (PIGF) and vascular endothelial growth factor (VEGF), are present. Altogether, these alterations seem to cause a decrease in NO levels and, thus, a state of maternal endothelial dysfunction, with hypertension development and consequential signs and symptoms of PE. Obesity is a multisystemic disease characterized by profound alterations in the endocrinological and immunological systems. Hyperleptinemia, hyperinsulinemia, dyslipidemia, and hypoadiponectinemia are some of the metabolic disturbances associated with obesity. Hyperleptinemia, hyperinsulinemia, and hypoadiponectinemia seem to be responsible for the perpetuation of an environment of profound inflammation. In addition, they seem to contribute to the enhancement of a state of endothelial dysfunction through reduction of the bioavailability and synthesis of NO, hence contributing to PE pathogenesis. Hyperleptinemia and hyperinsulinemia are also believed to contribute to an abnormal placentation process (*). Profibrinogen, plasminogen activator inhibitor 1, and angiotensinogen levels are increased in obese patients, and they seem to contribute to an overall hypertensive state in PE by increasing blood viscosity and enhancing renal sodium reabsorption. Agonistic autoantibodies to the angiotensin II type 1 receptor (AA AT1) have been shown to be produced by the placenta of pre-eclamptic patients and their action of elevating blood pressure may also be aggravated in obese individuals. The overall inflammatory milieu associated with obesity is also responsible for perpetuating the state of endothelial dysfunction by maximizing an environment of oxidative stress and by increasing the levels of sFlt-1. sFlt-1 levels, increased in obesity, are believed to be released by the placenta, adipose tissue, and circulation. Dyslipidemia also contributes to PE pathogenesis by stimulating cellular oxidative stress and the overall proinflammatory environment of obesity and PE and by compromising the normal invasion of the trophoblast. Created with BioRender.com: https://BioRender.com/e10f284 (accessed on 15 January 2025).

The dyslipidemia that can also accompany obesity seems to be of great importance in PE pathophysiology since increased circulation of fatty acids can culminate in the accumulation of these substances in the placenta, therefore perpetuating an environment of oxidative stress and inflammation, and thus compromising the development of the placenta, the invasion process of the trophoblast, and even the communication between the maternal and fetal environments [18,164]. A theory of a dysbiosis of the placental microbiome has even been proposed as a possible contributor of obesity in PE development, demonstrating its complex and multifactorial role in PE (Figure 4) [183].

## 8. Relation Between Obesity, Lipid Profile, and Adverse Pregnancy Outcomes

Obesity is often accompanied by an altered lipid profile, as demonstrated by the high levels of triglycerides, increased levels of LDL, and decreased levels of HDL that frequently coexist in obese patients [42]. This abnormal lipid profile is a result of an alteration in hepatic fat metabolism, in which the rates of lipogenesis are augmented [42]. These lipidic alterations frequently lead to the development of dyslipidemia and to the consequent accumulation of these lipids in several locations of the organism leading to, for instance, artery plaque buildup [27]. It should be noted, however, that amongst the set of physiological alterations in pregnancy is included a severe hyperlipidemia [16,98]. In fact, during a normal pregnancy, a gradual increase in triglycerides, HDL, and LDL is observed until the third trimester [98]. This physiological shift in the lipid profile is thought to be due to an increased synthesis of very low-density lipoproteins stimulated by the hyperestrogenemic environment of pregnancy and to a diminished capacity to eliminate lipoprotein triglycerides that is caused by dysfunction in one or both lipolytic enzymes [98]. Nonetheless, exaggeratedly increased cholesterol levels during pregnancy can be a trigger for the development of a state of endothelial dysfunction and can cause of damage to placental vasculature [16].

Lipid levels are important diagnostic tools in terms of aiding the identification of women who may have a higher risk of CVD, and since dyslipidemia constitutes one of the complications of obesity, as proposed by its syndromic coexistence in the MetS, they can potentially help identify obese women at risk of developing further complications [98]. Lipid profile has also been shown to be essential in predicting adverse pregnancy outcomes and overall complicated gestations [18]. This finding was supported by a study conducted in 2013 which demonstrated that high levels of cholesterol and ceruloplasmin during the 18th–20th gestational week could predict the development of PE [184]. A relationship between PE, dyslipidemia, and hypertriglyceridemia was also suggested by a study conducted in 1996 which confirmed the positive association between PE and an increase in triglycerides, free fatty acids, and malondialdehyde (MDA), a product of the lipid peroxidation process [185]. This increase in lipidic molecules was proven to decrease postpartum, suggesting that these lipidic alterations are intimately related to the placenta during gestation [185].

Hyperlipidemia is proposed to assume a crucial role in PE development through the facilitation of cellular oxidative stress and by potentiating an overall state of vascular impairment [98]. This was supported by a documented correlation between dyslipidemia and MDA in relation to the severity of PIH [98].

In pre-eclamptic patients, both the LDL/HDL ratio and the triglyceride levels are increased [16]. Thus, when combined with the hyperlipidemic state associated with obesity, these lipidic levels are further increased and, in that case, are responsible for an environment of enhanced inflammation and oxidative stress, facilitating the emergence of PE and its consequent signs and symptoms [146]. The abnormal lipidic state of both obesity and PE promote endothelial disfunction through several molecular mechanisms occurring at a vascular level [186]. The population of macrophages located in the intima of vessels can adopt pro- or anti-inflammatory properties depending on the ratio between HDLs and LDLs in circulation [16]. Therefore, an increased LDL/HDL ratio, specifically high levels of circulating LDLs and low levels of circulating HDLs, as seen in obese and pre-eclamptic individuals, can stimulate a shift towards the proinflammatory (M1) subtype of macrophages [16]. This is associated with the formation of foam cells located at the intima level of the vasculature and with the development of diffuse atherogenic processes [16]. The oxidized molecules of LDL circulating the luminal vessel are also known to contribute to increased levels of intercellular adhesion molecule 1 (ICAM-1) and vascular cell adhesion protein 1 (VCAM-1) and, consequently, to a state of activation and dysfunction of the endothelium [186]. Moreover, hypertriglyceridemia has been associated with a reduced release of prostacyclin by the endothelium contributing, hence, to the state of impaired vascular function associated with PE [98]. LDL levels also seem to correlate with an abnormal placentation process contributing, in addition, to the early disease mechanisms of PE [18]. In fact, hypertriglyceridemia combined with the free fatty acids released by obese individuals seem to contribute to this abnormal placentation process via regulation of the peroxisome proliferator-activated receptor-γ, the increased levels of which are associated with weakened trophoblast invasion [18].

These findings support the fact that for both early- and late-onset PE patients, an abnormal lipid profile has the ability to significantly influence the maternal lipidic metabolism and contribute to the emergence of PE [187]. Moreover, it can also potentially assume a role in the development of maternal disease and heightened maternal CVD risk in subsequent life stages, with these being possible complications seen in some women with PE [187].

The treatment of hyperlipidemia, such as hypertriglyceridemia, during pregnancy relies, primarily, on lifestyle modifications such as diet optimization and exercise [188]. Nonetheless, for patients with sustained and severe hypertriglyceridemia, such as triglyceride levels greater than 500 mg/dL, medical therapy may be considered [188]. The only group of drugs approved for use during pregnancy are bile acid sequestrants [189]. Omega 3 fatty acids, fibrates, nicotinic acid and ezetimibe are considered as options in selected cases but its use must be cautious given the fact that they are considered old category C by the FDA [188,189]. Statins are usually avoided given the unknown effects of this therapy for the fetus; however, the FDA recently removed the strict warning against statin use during pregnancy, allowing for individualized decision-making between patients and physicians [188,189]. New medications such as proprotein convertase subtilisin: kexin type 9 inhibitors, bempedoic acid, evinacumab, inclisiran, lomitapide, and mipomersen are being studied as potential future drugs for hyperlipidemia management during pregnancy [189].

In conclusion, dyslipidemia and the modifications of the maternal lipidic profile seem to act synergistically with the hormonal and immunological imbalances in AT in obese individuals, contributing to a setting of profound endothelial dysfunction and systemic inflammation correlating, therefore, with several adverse maternal and fetal outcomes [16].

## 9. Discussion

Maternal obesity numbers have been increasing worldwide during the last decades [3,5]. This is raising serious concern given the significant impact of obesity on adverse pregnancy outcomes [3]. Indeed, obesity constitutes a serious threat to both maternal and fetal health [3]. Obesity is commonly diagnosed using BMI, though the accuracy and relevance of BMI have been widely debated in recent years given its limitations in addressing the distribution of fat and overall CVD risk of each patient [7]. Moreover, BMI seems not to take into consideration physiological alterations in weight and volume related to pregnancy itself [7]. Several new tools have been proposed as valid indicators of obesity and its related complications; these include WC and waist/height ratio, amongst others [8,9]. Nonetheless, BMI is still used as a classification and diagnostic tool for pre-pregnancy, pregnancy, and postpartum periods [7]. Maternal obesity is related to a higher risk of conditions such as gestational diabetes and HDP [3,7]. HDPs are pregnancy-related complications with rising prevalence that pose a serious risk of maternal and fetal mortality [3,13,17]. HDP is a term referring to hypertension in the pregnancy period comprising four distinct conditions: GH, PE/eclampsia, chronic hypertension and chronic hypertension with superimposed PE [15,190]. The parallel and rising prevalence of obesity seems to contribute to overall increased numbers of HDPs driven by a higher number of obese or overweight women trying and managing to conceive [3,17].

The coexistence in obese individuals of conditions such as dyslipidemia, T2DM, IR, and arterial hypertension corresponds to the clinical translation of the set of endocrinological and immunological alterations that occur in the dysfunctional AT of these patients [9,30]. This set of pathological modifications related to an overall state of disrupted fat tissue homeostasis is referred to as adiposopathy, which seems to be the cause of an increased risk of CVD observed in some obese individuals [30,32,34]. This AT dysfunction is characterized by a state of low-grade inflammation, hyperleptinemia, hyperlipidemia, hypoadiponectinemia, and hyperinsulinemia [34,35]. Obesity, hence, is related to a dysmetabolic setting prone to the development of not only disease of the cardiovascular system but also of several other biological units such as the placenta [30,34]. Adiposopathy may, hence, partially unravel the complex pathophysiology of HDP and offer some insight into this group of disorders [3,164]. As a result, obesity management is of great importance to minimize the risk of CVD and to improve maternal and perinatal outcomes [2,3].

Obesity management in women of reproductive age, specifically in the pre-pregnancy, pregnancy, and postpartum periods, is based primarily on noninvasive approaches, such as improvement in dietary habits, and on exercise prescription [48,50,51]. When lifestyle strategies fail to produce satisfactory weight loss, an escalation of therapy may be considered for non-pregnant women [50]. Therefore, for women who adhere to proper contraceptive methods and have a BMI equal to or greater than 30 kg/m^2^ or a BMI equal to or greater than 27 kg/m^2^ and at least one obesity-related complication, pharmacotherapy may be instituted according to the patient’s individualized characteristics [54,56]. Bariatric surgery is also an option for women with a BMI equal to or greater than 40 kg/m^2^, or between 35 to 40 kg/m^2^ with comorbidities, when other therapeutic options are unsuccessful [54,57]. The ideal time for the implementation of weight loss strategies is exactly in the preconception or postpartum periods given the fact that these strategies are relatively safe approaches for both the mother and offspring and that these periods are characterized by fewer therapeutic restrictions [46,47]. Indeed, during pregnancy, the limitation of GWG to what is advised by the Institute of Medicine Guidelines—specifically, for obese women with a BMI equal or superior to 30 kg/m^2^ to between 5 and 9 kg—has shown to be beneficial [1,63]. The risk of small-for-gestational-age babies seems to be associated with weight loss, and thus it may not be recommended during the pregnancy period [1,47]. Notwithstanding, a healthy diet and physical activity should be recommended during gestation, with several studies demonstrating an association between lower risk of developing HDP and regular exercise, indicating the significant contribution of a healthy diet and physical activity to a healthy pregnancy [78,79,80]. Weight loss in the interpregnancy period has been associated with lower risk of HDP onset and, therefore, should be reinforced by clinicians [2,63].

Several studies conducted in recent years have confirmed the positive association between maternal obesity and HDP development in several populations [100,101,102,103,104,105,106,107]. Pre-pregnancy BMI was found to be related to higher mean arterial pressure levels and independently associated with higher blood pressure levels and HDP [113,114,115]. The evident connection between PIH and obesity is supported by several studies, with some authors having documented a three-times higher risk of HDP in obese individuals [116,117,118,119]. In fact, a linear association between an increase in pre-pregnancy BMI and PE was shown in 2003 by an author who demonstrated a doubling in PE risk with each five-to-seven unit increase in BMI [121]. Since then, recent studies have demonstrated the independent association between pre-pregnancy BMI and the development of PE and GH and have documented an exponential relationship between HDP risk and obesity class [100,109,114,127,128]. Maternal obesity has also been linked with the development of chronic hypertension and with progression to more severe states of hypertension, such as PE [125,126]. Furthermore, higher WC levels have proven to be related to a higher risk of GH and PE development, a further indication of the potential predictive role of this obesity indicator [100,129,130]. In addition, a high GWG is related to HDP risk, making obese women with an excessive GWG those at highest risk for the emergence of HDP [138,139,140,141]. The mechanisms of disease linking these two prevalent conditions are still unclear and presumed to be multifactorial [15]. The adiposopathy associated with obesity seems to increase the maternal susceptibility to placenta- released factors, and consequently, HDP development, through an enhancement of a state of generalized endothelial dysfunction [3,164]. This amplified state of endothelial dysfunction seems to be behind the PE pathogenesis in obese individuals [18,164]. In addition, this obesogenic milieu seems to contribute to PE emergence by aggravating the imbalance between proangiogenic and antiangiogenic factors associated with the latter condition, contributing to the state of endothelial dysfunction previously mentioned [161,165,167,168]. Hyperleptinemia and hyperinsulinemia have also been linked to an abnormal trophoblast invasion process, supporting the notion that the hormonal imbalances in obesity act through several pathways to contribute to PE onset [161,165,176]. Hyperlipidemia and, specifically, hypertriglyceridemia also seem to enhance the inflammation, oxidative stress, and thrombosis-prone setting of obesity, and may thus contribute to the development of HDP [16,100,191,192]. Simultaneously, enhanced activation of the SNS and of the RAAS seems to contribute to sodium and water retention and, hence, the development of hypertension in obese women [27,31,161]. These mechanisms may be behind the pathophysiology of other conditions in the HDP group such as GH and chronic hypertension [27,31,161]. Although several new studies have been uncovering the pathogenesis of HDP and the role of adiposopathy on its development, much remains unknown about this group of disorders [3,18,164]. Nonetheless, they appear to be linked with obesity through several mechanisms and by sharing a state of metabolic, hormonal, and endocrinological dysfunction, thus rendering maternal obesity management an essential intervention to improve the overall health of the mother and offspring [18,164,165].

The present study has, however, some limitations that should be taken into consideration. The heterogeneity of studies included in this review may make it difficult to draw consistent conclusions. Moreover, the quality of the studies included in this review may be variable and the lack of original data may compromise our findings.

Future controlled studies are essential to deepen our knowledge of this multifaceted group of disorders. These new investigations may unravel new pathways and pathophysiological links between obesity and HDP and may help uncover the complex pathophysiology of these conditions. Such advancements may pave the way for the development of new effective therapeutic options that can play a role in improving maternal and neonatal outcomes.

## 10. Conclusions

Obesity is an emerging condition that tends to occur with an exponentially increasing frequency in recent decades. The magnitude of obesity is furthermore amplified when combined with the fact that it is the leading disorder amongst women of fertile age. This should raise serious concern, since obesity is related to adverse pregnancy outcomes, with maternal and fetal mortality, and with a transgenerational propagation of obesity and CVD to the offspring. Moreover, maternal obesity is related not only with a higher risk of HDP, especially, PE and GH, but also with a higher risk of progression to more severe states of hypertension. Excessive GWG, frequently associated with maternal obesity, is also a risk factor for the development of HDP; this finding should motivate closer monitoring during obstetric consultations. The syndromic coexistence in obese patients of multiple conditions such as T2DM, IR, and dyslipidemia seems to act by inducing a milieu of endothelial dysfunction and inflammation that confers a greater susceptibility to the development of HDP. The role of obesity on PE pathogenesis is, indeed, complex and most likely multifactorial. In summary, the metabolic alterations in obesity may function independently or synergistically to enhance the pathological remodeling of the spiral arteries, amplify the secretion of soluble placental factors into the maternal bloodstream, and intensify the maternal systemic response to these soluble placental ischemia factors, facilitating the development of PE. Several questions remain unanswered, however, regarding the link between obesity and HDP and the ways in which these two complex conditions intertwine.

## Data Availability

Not applicable.

## Abbreviations

The following abbreviations are used in this manuscript:

BMI	body mass index
WHO	World Health Organization
PE	pre-eclampsia
GH	gestational hypertension
T2DM	type 2 diabetes mellitus
CVD	cardiovascular disease
IR	insulin resistance
HDP	hypertensive disorders of pregnancy
sBP	systolic blood pressure
dBP	diastolic blood pressure
GWG	gestational weight gain
MeSH	Medical Subject Heading
AT	adipose tissue
WC	waist circumference
MetS	metabolic syndrome
TNF-α	tumor necrosis factor α
NO	nitric oxide
RAAS	renin–angiotensin–aldosterone system
SNS	sympathetic nervous system
LDL	low-density lipoprotein
HDL	high-density lipoprotein
DASH	Dietary Approaches to Stop Hypertension
FDA	US Food and Drug Administration
EMA	European Medicines Agency
FIGO	Internal Federation of Gynecology and Obstetrics
NCD	noncommunicable diseases
PIGF	placental growth factor
PIH	pregnancy-induced hypertension
sFlt-1	soluble fms-like tyrosine kinase-1
VEGF	vascular endothelial growth factor
IPGs	inositol phosphoglycans
MDA	malondialdehyde
ICAM 1	intercellular adhesion molecule 1
VCAM 1	vascular cell adhesion protein 1
